# Supplementary material for: Is the Combination of Robot-Assisted Therapy and Transcranial Direct Current Stimulation Useful for Upper Limb Motor Recovery? A Systematic Review with Meta-Analysis
Source: Healthcare (Basel). 2024 Jan 29;12(3):337. doi: 10.3390/healthcare12030337 (PMC10855329; doi:10.3390/healthcare12030337)
Supplement: Supplementary file 1 [file healthcare-12-00337-s001.zip › healthcare-2713993-supplementary.pdf]

Supplementary Material

Supplementary Table S1. Search strategy.

Search keywords were based on the population (stroke) and constructs of interests (tDCS combined with robotic-assisted intervention); these included:

(tDCS OR transcranial direct current stimulation OR a-tDCS OR c-tDCS OR brain stimulation ORbrain stimulation techniques OR non-invasive brain stimulation)

AND

(robot* OR robotics OR robot assisted* OR robotic treatment OR robotic rehabilitation OR rehabilitation robot OR robot-assisted therapy* OR robot-based therapy OR Amadeo OR end effector OR end-effector OR exoskeleton OR exoskeleton device OR robotic exoskeleton)

AND

(stroke* OR hemiplegia OR hemiparesis OR cerebral vascular accident* OR cerebral vascular disorder OR CVA OR brain ischaemia OR brain infarction OR lacunar stroke OR brain injury OR pot-stroke)

AND

(upper limb OR upper extremity OR arm OR arm training OR wrist OR finger OR elbow OR shoulder)

Supplementary Table S2. Inclusion criteria.

Design

- Randomized clinical trials or crossover designs

Participants

- Adult age, > 18 years old
- People with subacute or chronic stroke
- No epilepsies or epileptic syndromes

Intervention

- Transcranial direct current stimulation (tDCS) combined with robot-assisted therapy (RAT) for upper limb

Outcome measures

- Primary: Deficit severity
- Secondary: Hand dexterity and spasticity

Comparison

- Real-tDCS combined with RAT for upper limb *versus* no/sham-tDCS intervention

Supplementary Table S3. Category data extraction.

Category	Data extraction
Methodology	Randomized; blinded; patient loss; experimental and control group; inclusion and exclusion criteria.
tDCS intervention	Area stimulated; intensity; timing (online u offline); time application; characteristics; tDCS design.
Robot-Assisted Therapy	Robot device; number of repetitions; time application.
Outcomes	Timing outcomes; basal outcome information; post outcomes information; first and secondary outcomes
Patients	Gender; allocated; n; hemispheric affected; area affected; age; time since stroke; stroke type.

Supplementary Table S4. GRADE System application: Classification of quality of evidence and strength of recommendation of the included studies.

GRADE Evidence				
	**Deficit severity**	**Hand dexterity**	**Spasticity**	**Activity**
**Test**	FMA UE	BBT	MAS	BI
**Nº of studies**	8	5	5	3
**Nº of comparations**	9	5	5	3
**Design**	RCT	RCT	RCT	RCT
**Risk of bias**	Not serious	Not clear	Not clear	Not serious
**Inconsistency (Heterogeneity)**	Low	Low	Low	Low
I^2	0%	0%	0%	2%
**Undirect evidence**	No	No	No	No
**Imprecision**	No	No	No	No
N Total	354	133	200	162
Optimal information size (OIS) for Effect size=0.3 (medium)	82	82	82	82
**Publication Bias**	Not clear	Not clear	No	No
**Índice LFK**	-2.15	2.62	1.11	0.52
**P Value Test of Begg**	0.71	0.46	0.8	1
**P Value Test of Egger**	0,36	0.18	0.59	0.7
**Effect size: SMD (95%CI)**	-0.09 [-0.31, 0.12]	0.12 [-0.22, 0.46]	0.04 [-0.24, 0.32]	0.66 [-1.82, 3.14]
**Large effect size (SMD>0.8)**	No	No	No	No
**Dose-response effect**	Not applicable	Not applicable	Not applicable	Not applicable
**Control for confounding factors**	High	High	High	High
**GRADE evidence level**	High	High	High	High

(- Level) = Down level evidence; (+ Level) = Up level evidence; SMD = Standardized mean difference.
FM-UE = Fugl-Meyer Assessment Upper Extremity; MAS = Modified Ashworth Scale; BBT = Box and Block Test; BI = Barthel Index.

Supplementary Table S5. PEDro criteria and scores for the included trials (n = 9).

Study	Random allocation	Concealed allocation	Groups similar at baseline	Participant blinding	Therapist blinding	Assessor blinding	< 15% dropouts	Intention to-treat analysis	Between-group difference reported	Point estimate and variability reported	Total (0 to 10)
**(Hesse et al., 2011)**	Y	Y	Y	Y	N	Y	Y	N	Y	Y	8
**(Morone et al., 2022)**	Y	Y	Y	Y	Y	Y	Y	N	Y	Y	9
**(Triccas et al., 2015)**	Y	Y	Y	Y	N	Y	Y	N	Y	Y	8
**(Straudi et al., 2016)**	Y	Y	Y	Y	N	Y	Y	N	Y	Y	8
**(Dehem et al., 2018)**	Y	Y	Y	Y	Y	Y	Y	N	Y	Y	9
**(Edwards et al., 2019)**	Y	Y	Y	Y	N	Y	Y	N	Y	Y	8
**(Mazzoleni et al., 2015)**	Y	Y	Y	Y	N	Y	Y	N	Y	Y	8
**(Mazzoleni et al., 2017)**	Y	Y	Y	Y	N	Y	Y	N	Y	Y	8
**(Mazzoleni et al., 2019)**	Y	Y	Y	Y	N	Y	Y	N	Y	Y	8

N = No; Y = Yes.

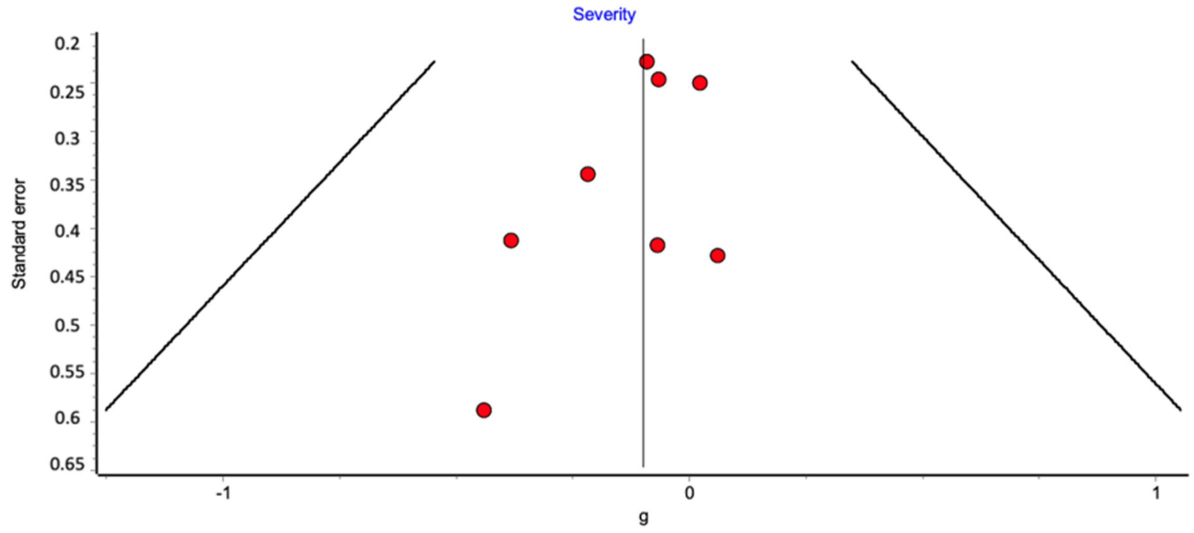

Funnel plot-Deficit Severity

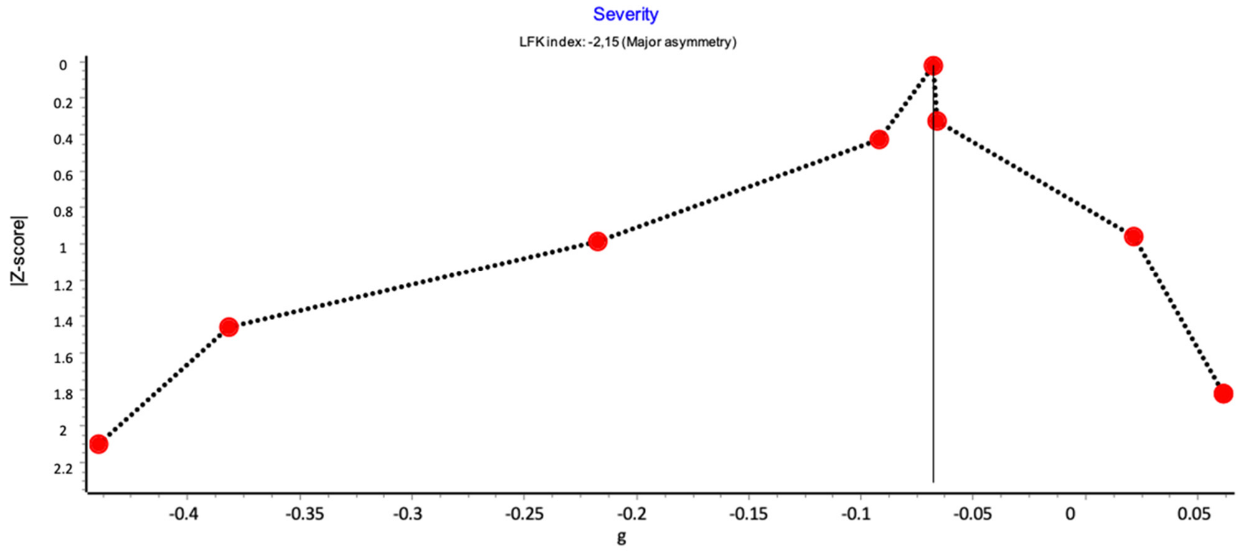

DOI plot-Deficit Severity

(B) Hand dexterity.

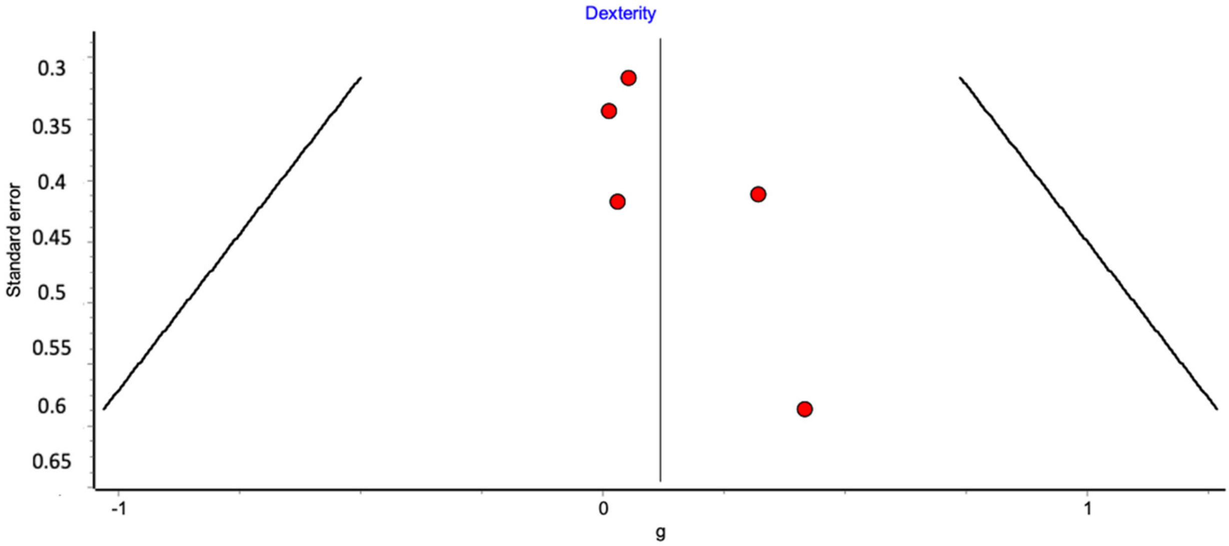

Funnel plot-Hand dexterity

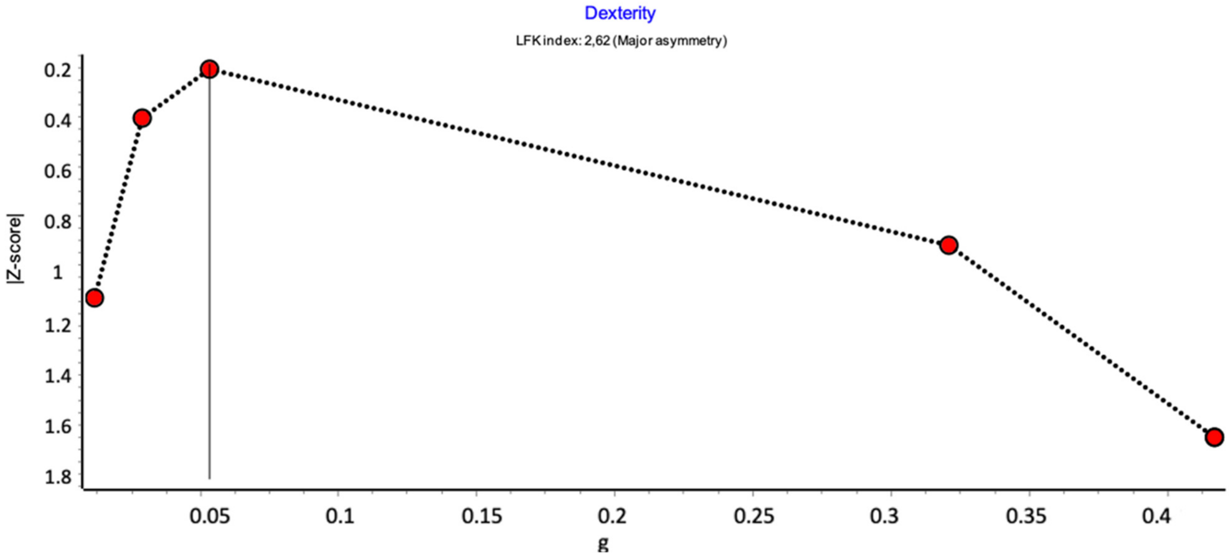

DOI plot-Hand dexterity

(C) Spasticity.

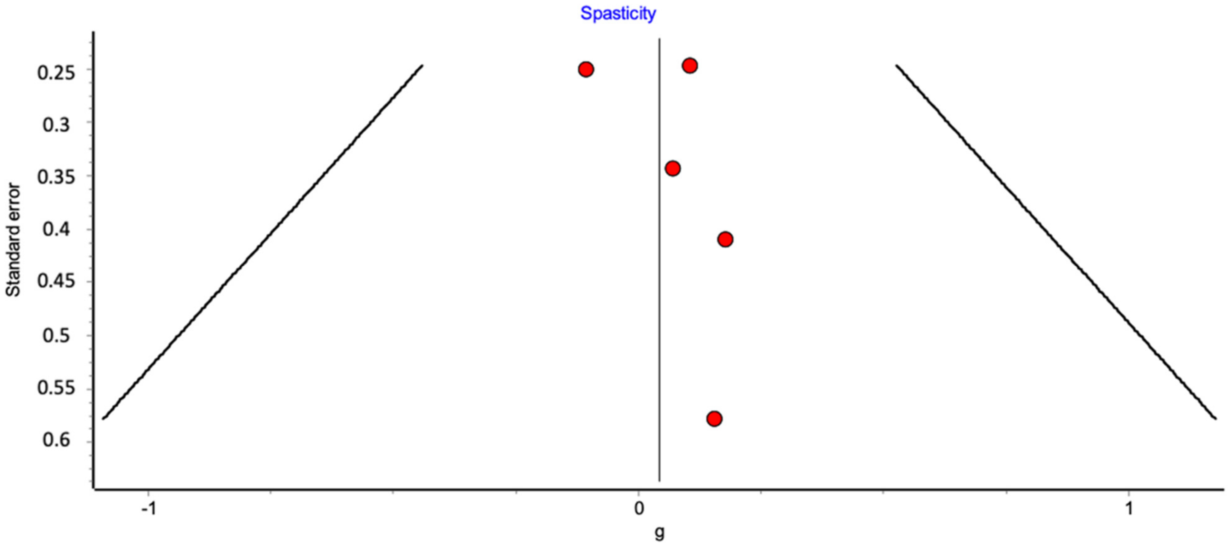

Funnel plot-Spasticity

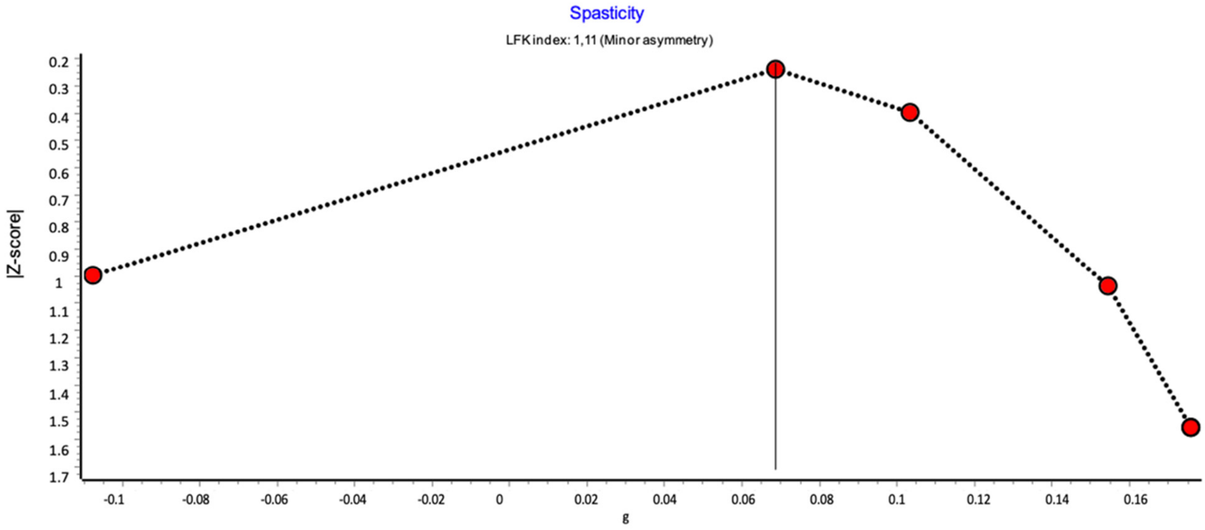

DOI plot-Spasticity

(D) Activity.

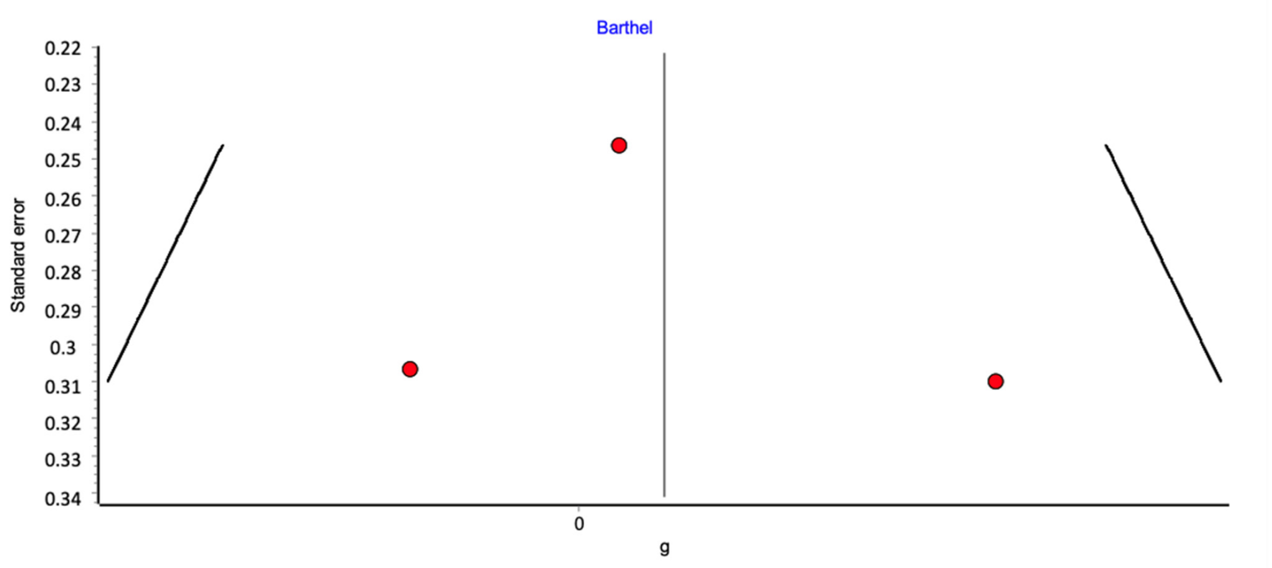

Funnel plot-Activity

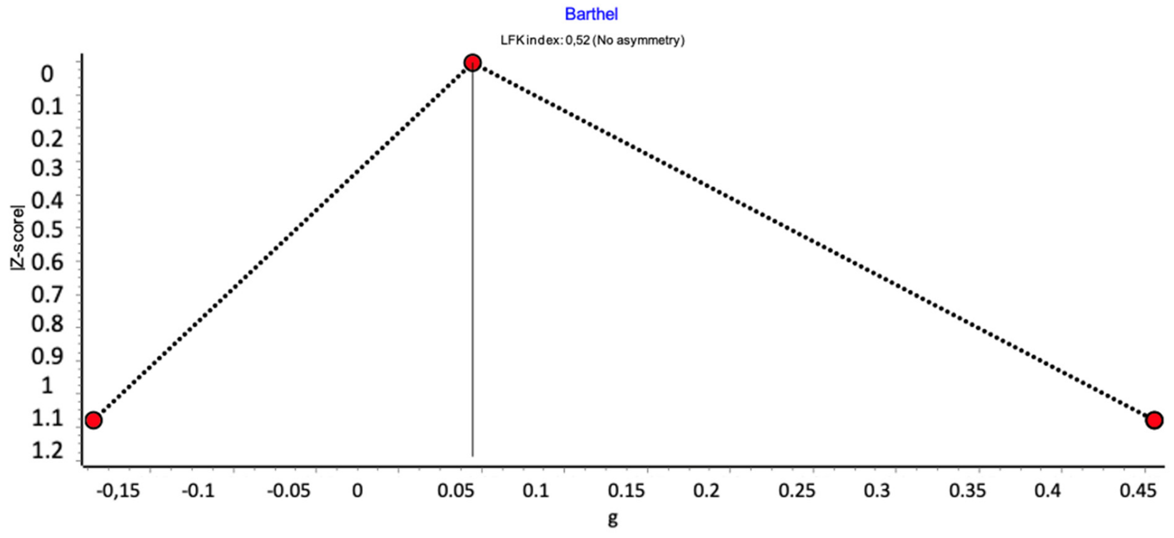

DOI plot-Activity

Supplementary Figure S1: (A) Deficit Severity.
